# GPR44 as a Target for Imaging Pancreatic Beta-Cell Mass

**DOI:** 10.1007/s11892-019-1164-z

**Published:** 2019-06-27

**Authors:** Olof Eriksson

**Affiliations:** 1 Science for Life Laboratory, Department of Medicinal Chemistry, Uppsala University, Dag Hammarskjölds väg 14C, 3tr, SE-752 37 Uppsala, Sweden; 2 Antaros Medical AB, Mölndal, Sweden

**Keywords:** Beta-cell imaging, Islet imaging, Beta-cell mass, GPR44, PET, Diabetes

## Abstract

**Purpose of Review:**

Quantitative markers for beta-cell mass (BCM) in human pancreas are currently lacking. Medical imaging using positron emission tomography (PET) markers for beta-cell restricted targets may provide an accurate and non-invasive measurement of BCM, to assist diagnosis and treatment of metabolic disease. GPR44 was recently discovered as a putative marker for beta cells and this review summarizes the developments so far.

**Recent Findings:**

Several small molecule binders targeting GPR44 have been radiolabeled for PET imaging and evaluated in vitro and in small and large animal models. ^11^C-AZ12204657 and ^11^C-MK-7246 displayed a dose-dependent and GPR44-mediated binding to beta cells both in vitro and in vivo, with negligible uptake in exocrine pancreas.

**Summary:**

GPR44 represents an attractive target for visualization of BCM. Further progress in radioligand development including clinical testing is expected to clarify the role of GPR44 as a surrogate marker for BCM in humans.

## Introduction

Loss of functional beta-cell mass (BCM) is a hallmark feature in both type I and type 2 diabetes (T1D/T2D). Our knowledge on the progressive change in BCM during development of diabetes is currently dependent on biopsy, usually obtained post-mortem. The datasets describing dynamic changes in BCM in pancreas are therefore often cross-sectional in nature. On the other hand, these are lacking the crucial longitudinal component, evidently needed given the remarkable variation (up to power of 10) in BCM in non-diabetic subjects [1].

Readily accessible plasma markers for beta-cell function, such as insulin, c-peptide, and HbA1c, are dependent on several downstream metabolic pathways and do not correlate well to BCM.

The possibility of using medical imaging technology to visualize and accurately measure human and animal BCM has been intensely explored during the last decade [2]. Positron emission tomography (PET) is of particular interest due to the high sensitivity and potential for quantification of this technology. Briefly, suitable molecules may be labeled with unstable nuclides which decay by emission of positrons, i.e., the antiparticle of the electron. When the positron encounters an electron, both particles are annihilated while generating two gamma photons, emitted in opposite directions, which can be detected by a PET scanner with reasonable temporal (seconds) and spatial (millimeters) resolution. The radiolabeled molecule can thus be traced inside a living organism, including humans, following administration. If the labeled molecule binds to a receptor with sufficient affinity and specificity, it is in theory possible to measure the receptor density in a tissue of interest.

Several targets restricted to the beta cells within the pancreas have thus been proposed as potential markers for BCM imaging. More targets than can be discussed under the scope of this review have been suggested [2], but some of the more prominent as well as those evaluated by human PET studies include the vesicular monoamine transporter 2 (VMAT2) [3], the glucagon like peptide-1 receptor (GLP1R) [4], the serotonin biosynthesis pathway [5], and the dopamine receptor subtype 3 (DRD3) [6]. These targets, although promising to variable degrees, all exhibit features complicating their use in the context of BCM imaging, such as species-dependent background expression in the acinar pancreas or expression in other endocrine cell subtypes [7–9]. Thus, it is of interest to identify and assess novel beta-cell restricted molecular targets to facilitate novel imaging approaches.

In addition to providing a novel technique for assessing the change in BCM in the development of metabolic disease, such imaging technology may also provide important new endpoints in pharmaceutical drug development. Given the residual remaining BCM in the pancreas in subjects with long-standing T2D [1], but also T1D [10, 11], the expansion of endogenous beta cells may be a possible anti-diabetic treatment. For example, GLP-1 agonism has been demonstrated to expand BCM in rodent models on group level by post-mortem pancreas analysis [12], but it is not trivial to demonstrate a similar effect in clinical studies. A sensitive BCM imaging marker could for the first time allow such a clinical endpoint, potentially opening up novel research areas also in human individuals. Also other types of beta-cell replacement technologies could benefit from a beta-cell imaging marker, for example, the relatively established intraportal transplantation of islets to subjects with T1D (where the treatment efficiency is far from optimal) [13] or emerging treatments such as transplantation of macro-encapsulated beta cells [14] or stem cell transplantation.

The current paper will review the development of current and future probes for PET imaging using the protein GPR44 as a marker for BCM.

## GPR44 Structure, Function, and Role as Therapeutic Target

**Fig. 1:**
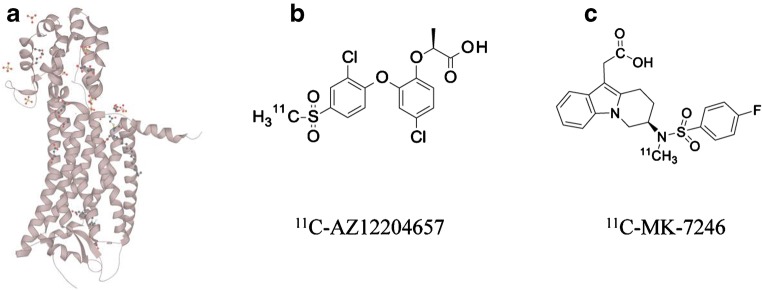
Structure of GPR44 (**a**) and two potential small molecule PET radiotracers for GPR44 (**b, c**)

GPR44, also known as prostaglandin D_2_ receptor 2 (DP_2_) or chemoattractant receptor-homologous molecule expressed on T-helper type 2 cells (CRTH2), is a transmembrane G-coupled receptor, activated by the endogenous prostaglandin D_2_. It consists of 7 transmembrane alpha helices; the crystal structure of GPR44 was recently published (Fig. 1a) [15]. GPR44 is a G protein-coupled receptor (GPCR) which acts negatively on cAMP production [16].

GPR44 is expressed on TH2 and eosinophil cells, and in this capacity it is involved in chemotaxis [17–19]. As the role of GPR44 in eosinophil migration was further elucidated, it emerged as a potential therapeutic target in inflammatory diseases including asthma [20]. Numerous small molecule GPR44 antagonists have therefore been developed and several of these have reached clinical testing [20]. Antagonists reaching early clinical phases include ramatroban and AMG863 (both non-selective GPR44 antagonists), OC000459, AZD1981, BI671800, and MK-7246 and more, while fevipriprant (QAW039) is currently in phase 3 [20].

In other tissues, the action of endogenous prostaglandin D2 (PGD_2_) on GPR44 in hair follicles has been linked to hair loss alopecia [21, 22], triggering research into a potential role of GPR44 antagonists as treatment for baldness [23].

## GPR44 Function in Beta Cells

The function of PGD_2_ in the islets of Langerhans was initially discovered already in the early 1980s. The presence of endogenous PGD_2_ in rat islets, as well as its effects on hormone secretion, including insulin, were demonstrated in a series of publications [24–27]. These early results clearly pointed to physiologically relevant expression levels of GPR44 in beta cells, but the discovery was largely forgotten or ignored for almost 30 years.

Interest in the GPR44 expression in human islet cells was rekindled following a proteomic screening for islet restricted receptors in 2012 [28, 29•]. The screen used the Human Protein Atlas to identify proteins with strong immunostaining in the islets of Langerhans, combined with no evidence of presence in the exocrine pancreas. Further evaluation of GPR44 localization as assessed by confocal microscopy in individual human islets demonstrated co-localization between the GPR44 antibody with insulin, but not glucagon or somatostatin, indicating that the expression was restricted to beta cells [29•, 30••].

Furthermore, islets positive for insulin immunostaining were positive also for GPR44 on pancreatic sections from healthy non-diabetic human subjects, but absent in insulin negative islet in sections from subjects with T1D. In a parallel transcriptomic screen of different pancreatic compartments, it was found that GPR44 mRNA expression was 26-fold higher in islets compared to exocrine tissue [31•].

Based on the observation that GPR44 is enriched in beta cells, it is reasonable to assume a role of this protein in their cellular function. Given that GPR44 suppresses cAMP production [16], it was hypothesized that PGD_2_ (whether produced locally or systemically) may, via agonism at GPR44, act as a break on insulin production in response to glucose [32•]. Consequently, antagonism at the GPR44 may increase insulin secretion. Additionally, dysregulation of PGD_2_ signaling or GPR44 activity in beta cells may be implicated in metabolic disease.

Skrtic et al. found that PGD_2_ is produced by stellate cells in human islets of Langerhans, especially in T2D, providing a potential mechanism for dysregulation of insulin secretion [32•]. Furthermore, PGD_2_ inhibits glucose potentiation of insulin secretion in human beta-cell lines and human islets, but importantly can be normalized by co-incubation with the GPR44 antagonist AZD1981. In spite of these promising in vitro results however, therapeutic doses of AZD1981 did not affect insulin secretion in individuals with T2D in a clinical cross-over study [32•]. Thus, the association of GPR44 in the in vivo regulation of the insulin secretory machinery and possible dysfunction in human diabetes is still uncertain.

Still, both in vitro and clinical studies point to the robust expression of GPR44 in beta cells, thus constituting a putative imaging marker specifically for beta cells, given the development of ligands suitable for radiolabeling.

## Development of GPR44 Imaging Ligands

As discussed above, several small molecule antagonisms have been developed for targeting of GPR44. However, ligands optimized for imaging applications have different requirements compared to those intended for therapeutic applications. For example, oral bioavailability is imperative for a drug candidate and constitutes an important feature for candidate selection, while this is largely unimportant for an intravenously administered PET ligand. Additionally, increased lipophilicity and associated increase in off-target binding may be inconsequential for a drug candidate, while non-specific in vivo binding in the PET situation may obscure any receptor-specific binding present in the tissue of interest.

AZD3825, a small molecule antagonist against GPR44, was developed by high throughput screening using a ^3^H-PGD_2_ displacement assay. AZD3825 is potentially suitable for imaging due to its IC_50_ in the nanomolar range and relatively low lipophilicity [31•]. The ligand was tritiated (^3^H-AZD3825) and used to assess GPR44 receptor density (*B*_max_) as well as affinity (*K*_d_) in human immortalized beta cells (EndoC-BH1), islet and exocrine tissue [31•]. Its affinity was in nanomolar range in all tissues, but *B*_max_ was > 45 and > 6 larger in beta cells and islets respectively compared to exocrine tissue. The possibility of visualizing a target by PET depends on the interplay between both number of available receptors in tissue, as well as the affinity. The relationship is often expressed as binding potential (BP) (Eq. 1), where BP > 2 is desired for sufficient signal in tissue. As evident from Eq. 1, low expression in tissue can be compensated for by increased affinity.

1 $$ BP=\frac{B_{\mathrm{max}}\ \left(\frac{pmol}{mL}\right)}{K_{\mathrm{d}}(nM)} $$

In beta cells, GPR44 density was measured as approximately 23 pmol/mL, while affinity was 1.2 nM, yielding a BP = 19.2. On the other hand, in exocrine cells, BP was 0.3. Rat beta cells (INS-1 cell line) did not exhibit significant expression of GPR44 indicating species variation. AZD3825 was thus identified as lead compound for development of a PET tracer, for isotopic radiolabeling with Carbon-11. Using AZD3825 as starting point, a demethylated precursor for AZ12204657 (Fig. 1a) was developed [30••].

AZ12204657 was shown to displace tritiated PGD_2_, as well as tritiated AZ12204657 itself, dose-dependently from membranes of HEK293 cells overexpressing human GPR44 [30••]. AZ12204657 also acts as an antagonist on GPR44 as it could inhibit the PGD_2_ signaling in human immortalized EndoC-BH1 beta cells [30••]. Demethylated AZ12204657 was then radiolabeled with Carbon-11 by addition of ^11^C-CH_3_I as described in detail previously (Fig. 1b) [30••].

Another approach for the identification of suitable PET imaging ligands for GPR44 has included radiolabeling of some of the drugs currently or previously in clinical trials. The upside of such an approach is that the ligands are presumably thoroughly characterized (affinity, lipophilicity, protein binding, etc.) as well as tested for toxicology. On the other hand, such ligands may not be optimal for imaging, with regard to lipophilicity etc. as discussed above. MK-7246, as an example, possesses an affinity and IC_50_ just above the nanomolar range [33–35].

Crucially, it also contains a fluorine nuclide, which potentially can be isotopically replaced by a Fluorine-18 and thus a preferable longer radioactive half-life (Fig. 1c). Demethylated MK-7246 was also radiolabeled by addition of ^11^C-CH_3_I, similar as for ^11^C-AZ12204657 [36••]. Fevipriprant is also potentially an attractive imaging ligand, given its high nanomolar affinity and its relatively low lipophilicity (logP = 2.3) but is so far unexplored in the imaging setting [37].

The use of radiolabeled peptides and biologics such as monoclonal antibodies (mAbs) for in vivo imaging has increased during the last decades. Several mAbs directed towards human GPR44 are available for different applications including immunofluorescent in vitro imaging. It is therefore conceivable to consider radiolabeling of mAbs also for GPR44 in vivo molecular imaging.

However, given the slow clearance of mAbs, several days of biodistribution and clearance are usually required from administration to achieve high-contrast imaging of tissues of interest. Antibodies are therefore often radiolabeled with radionuclides with radioactive half-life in the range of days such as Zirconium-89 (PET, *t*_1/2_ = 4 days), Indium-111 (SPECT, *t*_1/2_ = 4 days) or Iodine-124 (PET, *t*_1/2_ = 4 days). The substantial deposited radiation dose associated to these radionuclides does in practice preclude repeated PET or SPECT imaging in many patient groups and in healthy individuals or those with diabetes involved in research studies. In order to decrease the radionuclide half-life, and thus the absorbed dose, the biological half-life of the antibody must be decreased in some fashion, often by miniaturization of the construct. This can be achieved by, for example, using the binding domain, such as single chain variable fragments (scFv), thus in theory retaining excellent target affinity while reducing time for blood clearance. Other possibilities include directed protein engineering techniques including generations of nanobodies (relatively small camelid single domain antibodies) or affibody constructs (based on a 6.5 kDa peptide scaffold). Such small protein constructs display more rapid biodistribution often compatible with short-liver radionuclides such as Gallium-68 or Fluorine-18. However, raising, for example, affibodies towards the relatively small extracellular domain of GPR44 may pose an issue.

Based on the possibility of precursor synthesis, as well as convenient Carbon-11 by methylation, MK7246 and AZ12204657 have so far been evaluated in vitro and in vivo for GPR44 imaging.

## Imaging Results—In Vitro Approaches

The preclinical characterization of unlabeled MK7246 and AZ12204657 demonstrated high affinity and specificity to GPR44. Since both molecules were labeled isotopically by substitution of a stable Carbon-12 to a positron emitting Carbon-11 nuclide, the resulting PET tracers ^11^C-MK7246 and ^11^C-AZ12204657 should in theory behave in an identical manner as their respective unlabeled analogue (excluding any negligible kinetic isotope effects).

High islet-to-exocrine receptor binding ratio is required for any islet or beta-cell imaging agent, in order to yield sufficient contrast in imaging studies. The resolution of modern clinical PET scanners is around 3–5 mm and thus it is not currently possible to resolve individual islets of Langerhans in the pancreas. However, given sufficient contrast, the PET signal from each voxel will in theory represent the beta-cell density. The average beta-cell density (or average PET tracer concentration) multiplied with the total pancreatic volume will thus produce the total pancreatic BCM [2].

^11^C-AZ12204657 demonstrated strong binding to isolated human islets with a purity of 93% (Table 1). The binding was GPR44-mediated as it could be inhibited by co-incubation with GPR44 antagonist AZD3825 [30••]. The binding to pure exocrine tissue was a magnitude lower and non-specific, thus indicating a high islet-to-exocrine ratio. Islet homogenate binding studies have not yet been reported for ^11^C-MK-7246.

**Table 1:** Overview of in vitro and in vivo evaluation of PET radiotracers for GPR44

Assay		Species	Model	^11^C-AZ12204657	^11^C-MK7246
In vitro cell binding	Islet	Human	Healthy	High, receptor-specific	N/A
	Exocrine			Low, non-specific	N/A
In vitro autoradiography	Pancreas	Pig	Healthy	Pancreatic binding receptor-specific	N/A
			STZ T1D	No binding in pancreas	N/A
		Non-human primate		Pancreatic binding receptor-specific	N/A
		Human	Healthy	Binding in islets, receptor-specific	Binding in islets, receptor-specific
			T2D	Binding in islets, receptor-specific	N/A
			T1D	No binding in pancreas	N/A
In vivo targeting in mouse	Transplanted islets	Human	Healthy	Binding to islets, receptor-specific	N/A
In vivo targeting	Pancreas	Pig	Healthy	Receptor-specific	Receptor-specific
		Non-human primate	Healthy	Receptor-specific	N/A

In vitro autoradiography assays on human pancreatic sections have been performed for both ^11^C-AZ12204657 and ^11^C-MK-7246 as well as for tritiated ^3^H-AZ12204657. ^11^C-AZ12204657 demonstrated clear binding to focal areas of pancreas from non-diabetic donors and donors with T2D [30••, 38••]. The focal uptake was identical to the islets of Langerhans, as demonstrated by immunofluorescent staining for insulin on adjacent sections, again demonstrating a high islet-to-exocrine binding ratio. Importantly, no binding was seen in pancreatic sections lacking positive insulin staining. The focal binding patterns were GPR44-mediated (blockable by addition of AZD23825), in line with the results from the binding studies in isolated islets. Similar results were seen for ^3^H-AZ12204657 on human pancreatic sections from non-diabetic donors [30••]. ^11^C-MK-7246 also bound in clear focal patterns to human pancreatic sections from non-diabetic and T2D donors, which was likely receptor-mediated as it could be abolished by co-incubation with unlabeled MK-7246 added in excess [36••]. The focal patterns were dense especially in areas positive for insulin staining. Additional studies in T1D pancreas devoid of insulin positive islets as well as binding studies to islets and exocrine tissue should be performed for ^11^C-MK-7246 to verify these initial results.

## Imaging Results—In Vivo PET

Even if a PET tracer demonstrates excellent binding properties in vitro, there may be additional challenges to repeat such observations in vivo. In vitro assays are often designed to expose the tissue or receptor of interest directly to intact functional ligand at optimal conditions (correct buffer, temperature etc.). In the in vivo situation on the other hand, the PET tracer is administered peripherally, and is exposed to the organism’s blood and tissues as well as many metabolic and excretionary processes, before reaching the intended target where it may bind. Thus, it is imperative to demonstrate in vivo targeting in the tissue of interest of a PET tracer, also when the in vitro results are promising. Initial in vivo targeting is often assessed in small animal models such as mouse or rat. In the case of GPR44, rat and mouse pancreatic islets do not exhibit the same strong immunostaining for this protein as that seen in human [31•] or pig (not published). Accordingly, rat beta cell derived INS-1 cells showed negligible binding of ^3^H-AZD3825 [31•]. Thus, rat and mouse pancreas are not expected to provide a suitable model for GPR44 targeting PET tracers that translate to large animals and the clinical situation. A humanized mouse model can in this situation be generated, by transplanting human islets of Langerhans to immune-deficient mice. In a model where human islets where allowed to engraft in the kidney capsule, ^11^C-AZ12204657 demonstrated strong binding in the capsule containing islets, but negligible binding in the contralateral non-transplanted kidney capsule (Table 1) [38••]. Also in vivo the binding could be abolished by pre-treatment with 4 mg/g AZD3825.

In vitro autoradiography demonstrated focal binding patterns for ^11^C-AZ12204657 in pancreas from non-diabetic pig, consistent with immunohistochemical staining for insulin on adjacent sections [38••], and the previously demonstrated expression of GPR44 in pig islets (unpublished data). Importantly, beta-cell deficient pancreas sections from pigs treated by streptozotocin were negative for ^11^C-AZ12204657 binding. Accordingly, PET/MRI imaging with ^11^C-AZ12204657 in pig showed binding in pancreas, which could be competed away dose-dependently. Pre-treatment with 5 mg/kg GPR44 antagonist AZ8154 reduced the pancreatic binding by around 60%. Interestingly, significant ^11^C-AZ12204657 binding, blockable by on average 86%, was also seen in pig spleen, which was verified by in vitro autoradiography of pig splenic sections. The splenic binding is not expected to occur in humans in vivo, based on in vitro autoradiographic studies of human splenic sections [38••]. ^11^C-MK-7246 was evaluated in pigs by PET/CT, where significant binding similarly was seen in pancreas and spleen [36••]. The binding was reduced following pre-treatment with 1 mg/kg unlabeled MK-7246, by 66% in pancreas and 88% in spleen. It is interesting that the GPR44 receptor mediated in pancreas (60 vs. 66%) and splenic (86 vs. 88%) binding is so similar for the different PET tracers, indicating a similar mechanism of binding, as expected. The excretion of both tracers in pig was primarily through bile excretion into the intestines.

^11^C-AZ12204657 was also evaluated in cynomolgus non-human primates (NHP) by PET/CT. As in pigs, binding was demonstrated in both pancreas and to a lesser extent spleen, and the binding in both tissues could be abolished by pre-treatment by 1 mg/kg of GPR44 antagonist AZD3825 [38••]. The GPR44-mediated mechanism could be corroborated by in vitro autoradiography studies on pancreatic sections from NHP with and without blocking agent AZD3825, and co-staining for insulin [30••]. However, the strong signal from radioactive metabolites excreted into the intestines tended to cause spill in of signal into the pancreas, in particular the head and body. Thus, the NHP binding data is mainly based on ^11^C-AZ12204657 binding in the pancreas tail. This issue was likely due to the small size of the animals (around 5 kg) and small distance between intestines and pancreas in comparison with the PET/CT scanner resolution. The same issue was not seen in pigs (around 25 kg) and is not expected to cause any issues in potential future ^11^C-AZ12204657 scanning in humans.

Biodistribution studies was performed in rat by small animal PET/MRI scanning for ^11^C-MK-7246, to extrapolate the predicted radiation dose in humans [36••]. The excretion occurred mainly through the liver, biliary systems, and the intestines, as seen for both ^11^C-MK-7246 in pig and for ^11^C-AZ12204657 in both pig and NHP. The rat derived dosimetry is thus likely representative to human, even if the pancreas dose may be underestimated given the lack of specific binding to GPR44 in rodent islets. As expected, the absorbed dose associated with a Carbon-11 labeled molecule was low and compatible with several repeated PET/CT examinations annually [36••].

## Future Perspectives and Comparison with Other Beta-Cell Imaging Approaches

As outlined in the “Introduction,” there exist several different interesting putative molecular targets suggested as surrogate markers for beta cell or islet mass. The merits and challenges of each of the targets have been discussed in detail previously. Direct comparison with GPR44 targeting tracers may be premature and pending evaluation of ^11^C-AZ12204657 or ^11^C-MK-7246 in human studies.

It is important to note that the different performance of various imaging agents may represent different features of the endocrine pancreas and not simply the BCM. Serotonin marker ^11^C-5-hydroxytryptophan, for example, may be a tool indicative of total endocrine mass and provide value especially in combination with a beta-cell specific marker—i.e., to provide an endpoint, for example, for de-differentiation of beta cells into endocrine progenitor cells [5]. Similarly, VMAT2, GLP1R, and GPR44 imaging may all provide insights into different parts of beta-cell change in function or mass, depending on increased understanding of the role of respective receptor system in the endocrine pancreas.

Some general comments can be made regarding the different classes of ligand available for each target. VMAT2 imaging is currently dominated by Flurine-18 labeled small molecules which potentially enables important assays such as ex vivo autoradiography in animals, or optimized use of radiochemistry batches by scanning of several individuals or even shipping of PET tracer to remote sites. GLP1R imaging is currently performed by using different peptide constructs based on the Exendin-4 sequence to different degrees. Peptides can be radiolabeled with both Gallium-68 and Fluorine-18 but the clinical reality in the case of Exendin-based PET imaging is that Gallium-68 is commonly used. The practical logistical issues with Gallium-68 generally preclude the shipping of PET batches between sites and sharing of batches between patients. Additionally, there are dosimetric challenges associated with Gallium-68-labeled peptides, as the retention of the radioactive label in the renal cortex specifically makes the absorbed dose to the kidney a limiting factor. This is especially challenging in patient groups with different stages of renal dysfunction.

In this context, Carbon-11 labeled small molecules such as ^11^C-AZ12204657 and ^11^C-MK-7246 may provide an advantage given the limited deposited dose and the hepatic route of excretion. Carbon-11 has such a short radioactive half-life of only 20 min, that the associated radioactivity has largely disappeared only 2 h after administration. In combination with a beneficial dosimetry profile, this allows for individual patients to be examined several times during the course of a day, for example, to investigate drug targeting of the GPR44 receptor. However, the short half-life of Carbon-11 also precludes the possibility of sharing batches between patients or performing preclinical validation assays such as in ex vivo autoradiography studies. Thus, Fluorine-18 labeling of GPR44 targeting molecules is the next logical step in development. MK-7246 is in this framework especially promising given that it comprises a fluorine atom in a position theoretically available for radiolabeling (Fig. 1c).

An outstanding question for GPR44 targeting PET ligands are other uses besides imaging of beta cells, including its potential application as a marker for neuroendocrine tumors, especially insulinoma (i.e., beta-cell derived tumors). It is reasonable to expect a possible use in this field but currently no data is available on the GPR44 expression on insulinoma cells.

## Conclusion

Available studies indicate that GPR44 represents an attractive target for visualization of BCM. Several small molecule antagonists have been radiolabeled and evaluated up to large animal models. Further progress in radioligand development, including Fluorine-18 radiolabeling and clinical testing is expected to clarify their use as surrogate markers for BCM in human health and disease.
